# Comparison of Circulating, Hepatocyte Specific Messenger RNA and microRNA as Biomarkers for Chronic Hepatitis B and C

**DOI:** 10.1371/journal.pone.0092112

**Published:** 2014-03-18

**Authors:** Xiaonan Zhang, Zhanqing Zhang, Fahui Dai, Bisheng Shi, Liang Chen, Xinxin Zhang, Guoqing Zang, Jiming Zhang, Xiaorong Chen, Fangxing Qian, Yunwen Hu, Zhenghong Yuan

**Affiliations:** 1 Shanghai Public Health Clinical Center, Key Laboratory of Medical Molecular Virology at Shanghai Medical College, Fudan University, Shanghai, China; 2 Department of Viral Hepatitis, Shanghai Public Health Clinical Center, Fudan University, Shanghai, China; 3 Institute of Infectious and Respiratory Diseases, School of Medicine, Shanghai Jiaotong University, Ruijin Hospital, Shanghai, China; 4 Shanghai Sixth people’s hospital, Shanghai Jiaotong University, Shanghai, China; 5 Department of Infectious Diseases, Huashan Hospital, Fudan University, Shanghai, China; 6 Department of Chinese Traditional Medicine, Shanghai Public Health Clinical Center, Fudan University, Shanghai, China; 7 Department of Infectious Disease, Shanghai Changning Center Hospital, Shanghai, China; National Institute for Viral Disease Control and Prevention, CDC, China, China

## Abstract

Circulating microRNAs have been widely recognized as a novel category of biomarker in a variety of physiological and pathological conditions. Other reports revealed that fragments of organ specific messenger RNAs are also detectable in serum/plasma and can be utilized as sensitive indicators of liver pathology and cancer. In order to assess the sensitivity and reliability of these two class of RNAs as marker of hepatitis B or C induced chronic liver disease, we collected plasma samples from 156 chronic hepatitis B or C patients (HBV active n = 112, HBV carrier n = 19, hepatitis C n = 25) and 22 healthy donors and quantified their circulating mRNA for albumin, HP (haptoglobin), CYP2E1 (cytochrome P450, family 2, subfamily E) and ApoA2 (Apolipoprotein A2) in conjunction with microRNA-122, a well established marker for acute and chronic liver injury. We found that plasma microRNA-122 level is significantly elevated in patients with active HBV but not in HBV carriers. Furthermore, microRNA-122 is not elevated in HCV patients even though their median serum alanine aminotransferase (sALT) was three fold of the healthy donors. Nevertheless, circulating mRNAs, especially albumin mRNA, showed much more sensitivity in distinguishing active hepatitis B, hepatitis B carrier or HCV patientsfrom healthy control. Correlation and multiple linear regression analysis suggested that circulating mRNAs and miRNAs are much more related to HBsAg titre than to sALT. Immunoprecipitation of HBsAg in HBV patients’ plasma resulted in enrichment of albumin and HP mRNA suggesting that fragments of liver specific transcripts can be encapsidated into HBsAg particles. Taken together, our results suggest that hepatocyte specific transcripts in plasma like albumin mRNA showed greater sensitivity and specificity in differentiating HBV or HCV induced chronic liver disease than microRNA-122. Circulating mRNA fragments merit more attention in the quest of next generation biomarkers for various maladies.

## Introduction

Circulating nucleic acids in human peripheral blood has been increasingly acknowledged as indicators for a variety of physiological and pathological conditions including liver injury induced by hepatotoxic agents and viral hepatitis [1]. Although the quantity of free nucleic acids is generally very low in healthy and diseased subjects, the amplifiable nature and a plethora of quantification methodologies for these molecules facilitate its clinical application. In recent years, the idea that circulating microRNAs (miRNAs) can be sensitive markers for various maladies has been widely embraced. Indeed, quite a number of reports asserted that plasma miRNAs are excellent indicators for diseases ranging from acute liver injury [2], [3] to various malignancies [4], [5]. Meanwhile, fragments of messenger RNAs in plasma/serum were also found to reflect acute liver injury caused by hepatotoxic compounds [6], [7] and liver pathologies induced by hepatitis B virus [8].

Here, we aimed to employ a point-to-point evaluation of these two groups of marker in hepatitis B and hepatitis C virus induced liver disease. For microRNA, miRNA-122 was selected since it has been independently confirmed as a reliable indicator for liver injury caused by hepatoxic agents [2], [3] and hepatitis B virus [9], [10]. For mRNAs, albumin mRNA was one of the most abundant liver specific transcript and was shown to be induced in plasma in both chemically [6], [7] and virally induced hepatitis [8]. In addition, we also included transcripts for CYP2E1 (cytochrome P450, family 2, subfamily E), APOA2 (Apolipoprotein A2) and HP (haptoglobin) based on their tissue specificity and high abundance in hepatocytes.

## Materials and Methods

### Patients and specimens

A total of 178 participants from Shanghai Public Health Clinical Center, Ruijin Hospital, Shanghai Sixth people’s hospital, Huashan Hospital and Shanghai Changning Center Hospital were recruited in this study. Among them, 131 were HBV surface antigen positive, 25 were HCV RNA positive and 22 were healthy volunteers. All the HBV patients were negative for HCV antibody and all the HCV patients had >1000 copies/ml HCV RNA and were HBsAg negative. All the HBV and HCV patients were chronic hepatitis B patients without liver cirrhosis or hepatocellular carcinoma. The HBV patients were further divided into two groups (HBV active and HBV carrier) based on their HBVDNA and sALT level. Subjects in HBV active group (n = 112) had HBVDNA over 500 copies/ml irrespective of sALT level, subjects in HBV carrier group had positive HBsAg, undetectable HBVDNA (<500 copies/ml) and normal sALT (<40 U/L). The healthy volunteers were tested negative for HBsAg and HCV antibody with a normal sALT (<40 U/L). Their detailed basic characteristics were listed in Table 1. Hepatitis B virus surface antigen (HBsAg) and hepatitis B virus e antigen (HBeAg) were measured by Abbott AXSYM HBsAg (normal: 0–2S/N) and HBeAg by 2.0 MEIA kit (normal: 0–1.0S/CO) (Abbott Laboratories). HBV DNA was measured by a quantitative real-time PCR kit (Qiagen, Shenzhen, China) with >500 copies/ml as positive. HCV RNA was quantified by quantitative one-step RT-PCR kit (Qiagen, Shenzhen, China) with >1000 copies/ml as positive.

**Table 1:** Basic characteristics of enrolled individuals.

Clinical Variables	Healthy control	Hepatitis C	Hepatitis B carrier	Hepatitis B Active
	(n = 22)	(n = 25)	(n = 19)	(n = 112)
*Age-y* Median (Range)	43 (22–58)	51 (19–66)	42 (31–56)	33 (14–65)
*Gender*				
Male (%)	63.6	68	73.7	58
Female (%)	27.3	28	26.3	27.7
Missing (%)	9.09	4	0	14.3
ALT, median (range)	24.5 (16–43)	75 (17–204)	26 (19–39)	115 (16–2441)
U/L				
HBVDNA	Und	Und	Und	6.25 (2.72–7.7)
(log10 copies/ml)				
HCV RNA	Und	6.04 (3.60–7.37)	Und	Und
(log10 copies/ml)				
*HBsAg*				
Positive	0	0	19	112
Negative	22	25	0	0
HBsAg titre (IU/ml)	ND	ND	792 (382-2202)	3986 (829-22091)
Median(interquartile range)				

### Ethics statement

The study was approved by the institutional ethics review board of Shanghai public health clinical center. All patients and volunteers from whom specimens were obtained provided written informed consent.

### Sample collection and processing

Five milliliters of peripheral blood was collected into EDTA anticoagulent tubes. All blood samples were kept at 4°C. After centrifugation at 2000rpm for 5 minutes, plasma were collected, aliquoted and stored at –80°C until use.

### RNA extraction, reverse transcription and quantitative PCR

Plasma samples were thawed and centrifuged at 15,000 g for 10min at 4°C in order to precipitate microparticles. RNA was extracted with Trizol LS reagent (Invitrogen) from 200 μl of plasma supernatant according to the manufacturer’s instructions. To improve precipitation of small RNAs, supernatant from chloroform extraction were mixed with equal volume of isopropanol and stored at –80°C overnight. The precipitate were then recovered by centrifuged at 15000 g at 4°C for 10 minutes and washed with ice cold 85% ethanol. After solubilization in RNase free water, RNA samples were immediately reverse transcribed with reverseTra Ace (Toyobo, Japan) using Bulge-Loop RT primers for miRNA-122 (RiboBio, Shenzhen, China) and reverse primers for albumin, HP, CYP2E1, ApoA2 qPCR. The qPCR primer sequences are: ALB forward, TCTCTTTAGCTCGGCTTATTCC, ALB reverse, TCTTTAAACCGATGAGCAACCT; HP forward, TGCGATATCCGTGACATCAT, HP reverse, ACTGCTCTTCCAGAGGCAAG; CYP2E1 forward, CCCAATCACCCTGTCAATTT, CYP2E1 reverse, GACCACCAGCACAACTCTGA; ApoA2 forward, TTAACCAGTTCCGTTCCAGC, ApoA2 reverse, AGGTCAAGAGCCCAGAGCTT. Quantification of mRNA and miRNA were performed on a ViiA7 Real-Time PCR system (Applied Biosystems) using Thunderbird SYBR qPCR mix (Toyobo, Japan). For each marker, all the 178 cDNA samples were amplified in duplicate in a 384 well plate in order to avoid inter-batch variations. Melting curve analysis was performed following 45 cycles of amplification in each run. Reactions with abnormal melting curve were manually corrected as undetectable. The relative quantity of each gene were calculated using a standard 2^-ΔCt^ method.

### Immunoprecipitation of HBsAg and quantification of mRNA/miRNA

HBsAg positive plasma samples (100–200 ul) were diluted five fold in PBS and precleared with Protein A/G plus agarose beads (Santa Cruz). After centrifugation, supernatant was incubated with a monoclonal antibody against HBsAg for 2 hour at 4°C, Protein A/G plus agarose beads were then added to capture the HBsAg-antibody complex and washed with PBS three times. After immunoprecipitation (IP), an aliquot of Post-IP supernatant were saved and HBsAg was quantified along with Pre-IP sample using an HBsAg ELISA kit (Kehua biotech, Shanghai, China). miRNA-122 and mRNAs from Pre-IP, Post-IP samples and precipitated beads were extracted using Trizol LS reagent and quantified as described above.

### Statistical analysis

Statistical analyses were performed with PASW software (version 18, SPSS Inc). Plasma concentrations of mRNA and miRNA were compared using Mann-Whitney U-test with Bonferroni correction for multiple comparisons. Correlations among sALT and plasma markers were determined by the Spearman rank correlation. Multiple linear regression analysis was employed to evaluate clinical correlates of mRNA or miRNA. Area under the ROC curve (AUC) was used as an index for the performance of each marker.

## Results

### Concentration of plasma mRNA/miRNA in healthy and chronic HBV/HCV patients

**Figure 1:**
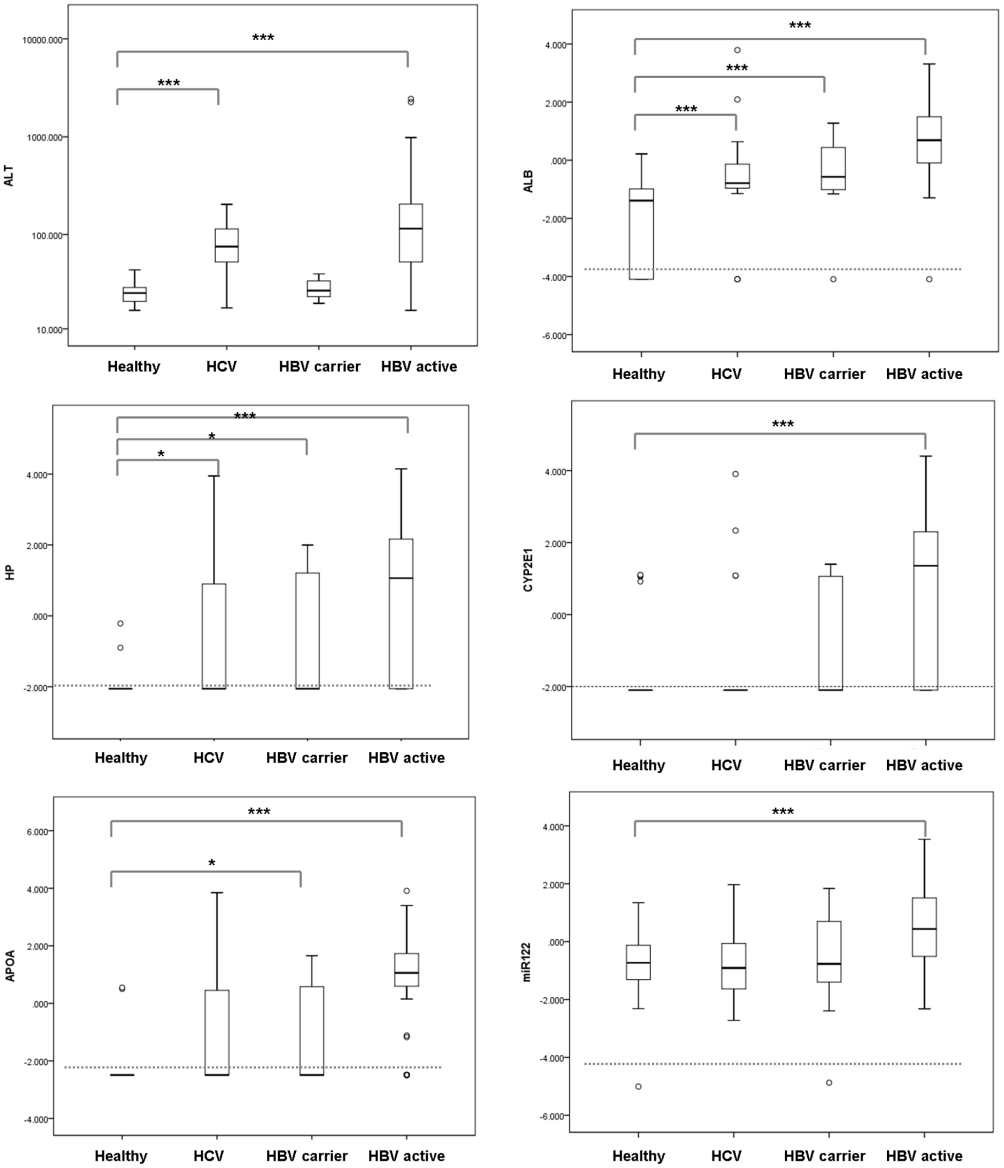
Quantification of plasma mRNA and miRNA-122. The sALT levels (A) of in healthy volunteers, HCV patients, HBV carriers and active replicating HBV patients and their plasma albumin(B), HP (C), CYP2E1(D), APOA2 (E) mRNA and miRNA-122 (F) levels were quantified and plotted The upper and lower limits of the boxes and the horizontal line within the boxes indicate the 75th and 25th percentiles and the median, respectively. The whisker caps indicate the 90th and 10th percentiles. Outliers are marked as open circles.

We quantified the abundance of messenger RNAs for albumin, HP, CYP2E1 and APOA2 mRNA in conjunction with miRNA-122 in 131 HBV, 25 HCV patients and 22 healthy donors by qRT-PCR. miRNA-122 was chosen as miRNA marker for liver disease because it is highly abundant in hepatocytes and is repeatedly reported to reflect liver damage [2], [3]. For mRNA markers, albumin, HP (haptoglobin), CYP2E1 (cytochrome P450, family 2, subfamily E) and ApoA2 (Apolipoprotein A2) were selected due to their abundance and specific expression in hepatocytes. HBV patients were further divided into HBV active (n = 112) and HBV carrier (n = 19) group based on their HBVDNA and sALT level as described in Material and Methods. The degree of liver damage as usually indicated by alanine aminotransferase was illustrated in Figure 1A. The HCV group and HBV active group showed abnormal sALT (p<0.001) whereas HBV carrier group has sALT level comparable to healthy control.

Plasma albumin mRNA was detected in 163 of the 178 samples (91.6%) with negative reading in 11 healthy donors, 4 HCV patients, 1 HBV carrier and 1 HBV active patient. The percentage of positive reading for the other markers are, HP 53.9%, CYP2E1 51.7%, APOA2 62.4% and miR-122 98.9%. We found that, overall, these five markers exhibited strong correlation as indicated by a correlation matrix (Table S1) with R values ranging from 0.685 (APOA2 v.s. miR-122) to 0.858 (albumin v.s. HP).

When comparing HBV active patients with healthy control, we found that all of these five markers showed significant elevation (p<0.0001 for albumin, HP, CYP2E1, APOA2 mRNA and p = 0.0003 for miR-122, see Table 2 and Fig 1B-F). In addition, albumin mRNA exhibited much higher fold change (562 fold of median value in healthy control) than miR-122 (14.79 fold). The difference between healthy control and HBV carrier group was much less obvious with only albumin, HP and APOA2 mRNA reaching statistical significance (Table 2, Fig1B-F). Among them, albumin mRNA exhibited the most distinction (6.61 fold of median value in healthy control, p = 0.0006) whereas miRNA-122 was not induced. Interestingly, in HCV group, with median sALT three fold of the healthy donors (75 U/L v.s. 24.5 U/L, Table 1), no obvious increase of miRNA-122 was observed, only mild elevation in albumin mRNA (p = 0.004) and HP mRNA (p = 0.05) was documented (Table 2). The lack of up-regulation in miRNA-122 and other mRNA markers in HCV patients hinted that these molecules may not be passively released into circulation after liver injury and the etiology of viral hepatitis strongly influences their abundance.

**Table 2:** Quantification of plasma mRNA and miRNA.

Concentration of Plasma biomarker	Healthy control	Hepatitis C	P^a^	Hepatitis B Carrier	P^b^	Hepatitis B Active	P^c^
(relative copy number, logarithmic)	(n = 22)	(n = 25)		(n = 19)		(n = 112)	
Albumin mRNA	–1.39 (–4.09-–0.98)	–0.79 (–1.04-–0.11)	**0.004**	–0.57 (–1.02-0.73)	**0.0006**	1.36 (–0.12-1.50)	**<0.0001**
							
HP mRNA	–2.06 (–2.06-–2.06)	–2.06 (–2.06-0.90)	**0.05**	–2.06 (–2.06-1.21)	**0.02**	1.06 (–2.06-2.19)	**<0.0001**
							
CYP2E1 mRNA	–2.10 (–2.10-–2.10)	–2.10 (–2.10-–2.10)	>0.05	–2.10 (–2.10-1.07)	>0.05	1.36 (–2.10-2.31)	**<0.0001**
							
APOA2 mRNA	–2.49 (–2.49-–2.49)	–2.49 (–2.49-–0.48)	>0.05	–2.49 (–2.49-0.59)	**0.01**	1.06 (0.59-1.75)	**<0.0001**
							
miRNA–122	–0.73 (–1.32-–0.09)	–0.91 (–1.70-–0.09)	>0.05	–0.77 (–1.41-0.75)	>0.05	0.44 (–0.53-1.52)	**0.0003**
							

Data are presented in median (interquartile range). All the P values were calculated using Mann-Whitney U test with Bonferroni correction for multiple comparisons (healthy as control).

a , Comparison between Hepatitis C and healthy control.

b , Comparison between Hepatitis B carrier and healthy control.

c , Comparison between Hepatitis B active and healthy control.

### ROC curve analysis

**Figure 2:**
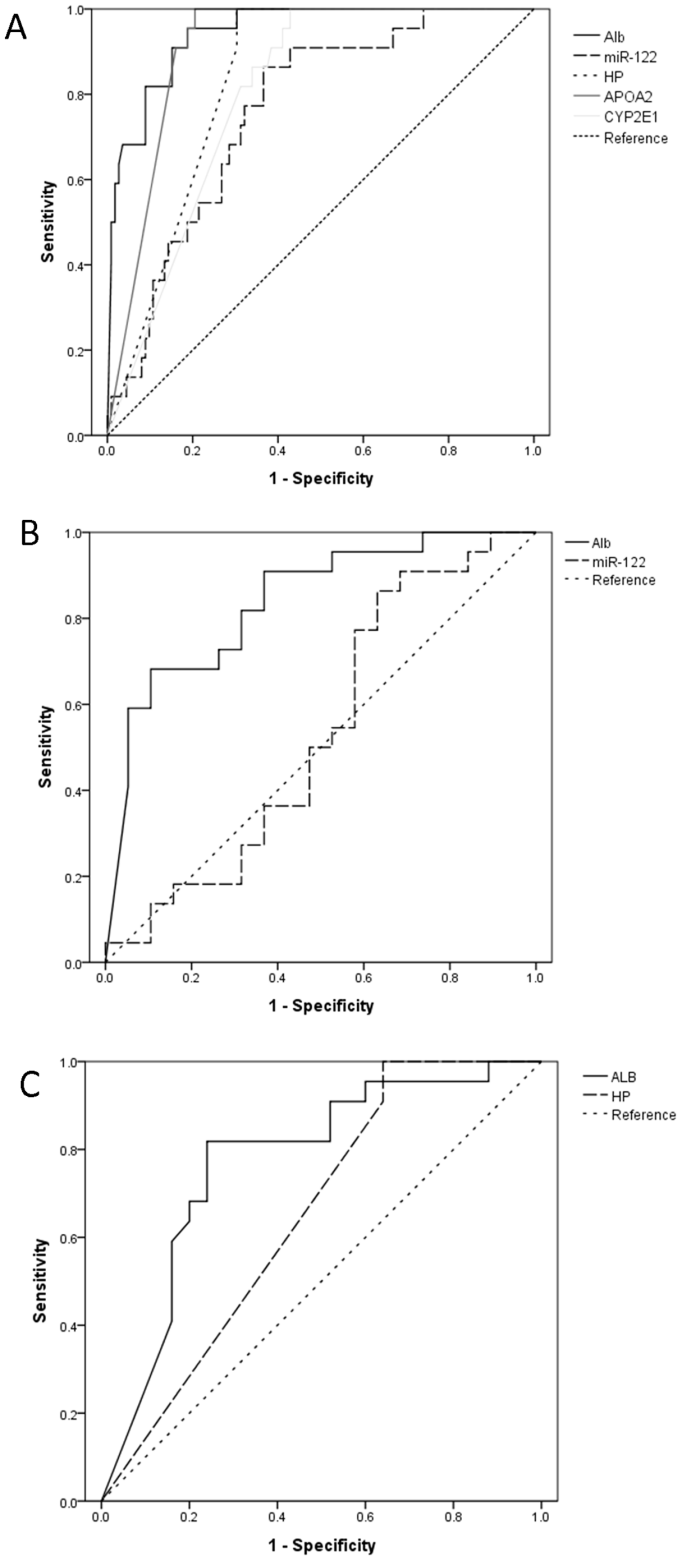
ROC curve analyses. ROC curve of circulating RNAs for the differentiation of HBV active group (A), HBV carrier group (B) and HCV group (C) from healthy control.

We next evaluated the sensitivity and specificity of mRNA/miRNA markers in distinguishing HBV/HCV infection from normal condition. Receiver operator curve analysis was performed and area under curve (AUC) was calculated for each marker against HBV active, HBV carrier and HCV group (Table 3, Figure 2). For HBV active group, all the markers showed positive results with AUC ranging from 0.762 (miR-122) to 0. 945 (albumin mRNA, Figure 2A). For HBV carriers, however, only albumin mRNA was found to be a reliable indicator (AUC, 0.839, 95%CI, 0.715–0.962) whereas miR-122 displayed no predictive value (AUC, 0.533, 95%CI 0.348–0.719, Figure 2B). Similar results were found in HCV group in which, marginal sensitivity (AUC, 0.774, 95%CI, 0.635–0.913) was found in albumin mRNA but not in the other markers. Collectively, albumin mRNA exhibited much greater differentiation ability than miR-122 in all three disease groups.

**Table 3:** Area Under Curve (AUC) analysis of plasma biomarkers.

Plasma biomarker	HBV active v.s. healthy	HBV carrier v.s. healthy	HCV v.s. healthy
Albumin mRNA	**0.945 (0.904**–**0.985)**	**0.839 (0.715**–**0.962)**	**0.774 (0.635**–**0.913)**
HP mRNA	**0.834 (0.767**–**0.901)**	**0.684 (0.514**–**0.855)**	0.651 (0.494–0.808)
CYP2E1 mRNA	**0.801 (0.726**–**0.877)**	0.629 (0.455–0.804)	0.496 (0.329–0.663)
APOA2 mRNA	**0.909 (0.861**–**0.957)**	**0.705 (0.540**–**0.872)**	0.601 (0.237–0.763)
miRNA-122	**0.762 (0.666**–**0.857)**	0.533 (0.348–0.719)	0.500 (0.333–0.667)

Area Under Curve (AUC) and 95% confidence interval in brackets was listed. AUCs with significant predictive values were in bold.

### The relationship between sALT, viral load, HBsAg titre and plasma mRNA/miRNA level

**Figure 3:**
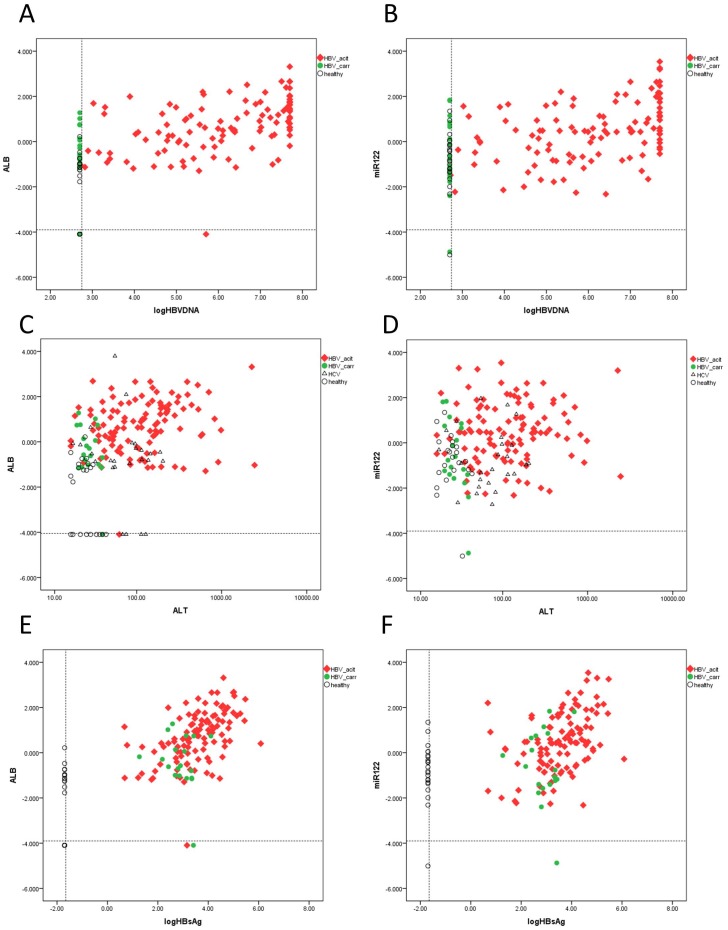
The relationship between sALT, viral load, HBsAg titre and plasma mRNA/miRNA level. Correlation plots between LogHBVDNA and albumin mRNA (A), between LogHBVDNA and miRNA-122 (B); between ALT and albumin mRNA (C), between ALT and miRNA-122 (D), between LogHBsAg and albumin mRNA (E), between LogHBsAg and miR-122 (F) were illustrated. The dashed lines indicate limit of detection.

Since previous analysis suggested that sALT level is not the sole factor associated with upregulation of plasma mRNA/miRNA markers. We further analyzed whether other factors (HBVDNA, HCVRNA, HBsAg titre, Bilirubin, alkaline phosphatase and γ-glutamyl transpeptidase) are related with these circulating molecules. Indeed, we observed that in HBV patients, HBV DNA level and HBsAg titre are actually much more related to these markers (r = 0.436–0.635, p = 6.06E-16—2.37E-7 for HBVDNA, r = 0.452–0.615, p = 1.19E-14—8.76E-8 for HBsAg, see Table 4, Figure 3), in terms of correlation coefficient and statistical significance, than sALT level (r = 0.204–0.369, p = 3.18E-7-0.006, see Table 4, Figure 3C-D). Indeed, after splitting HBV active group based on sALT level (< = 40 U/L v.s. >40 U/L) none of the markers exhibited notable difference (data not shown) which indicate that HBV viral load rather than alanine aminotransferase are the major determinant of their upregulation. For HCV patients, only albumin (r = 0.426, p = 0.003) and HP mRNA (r = 0.408, p = 0.005) showed barely significant association. We further attempted to establish a multiple linear regression model for albumin mRNA in HBV patients. sALT, HBsAg titre, total bilirubin, direct bilirubin, alkaline phosphatase and γ-glutamyl transpeptidase were included. HBVDNA was not included since it was highly correlated with HBsAg level (r = 0.761, p = 2.92E-29). It was observed that logHBsAg was the only independent variable (p = 2.83E-17) whereas LogALT did not reach statistical significance (p = 0.062, Table 5). For comparison, the same analysis was done on miR-122. Again, logHBsAg was found to be the only linear regression factor (p = 4.12E-6, r = 0.276, 95%CI, 0.162–0.390). Thus, correlation and multiple linear regression analyses strongly suggested that circulating mRNA/miRNA markers like albumin and miR-122 are more associated with HBsAg titre/HBVDNA than with sALT.

**Table 4:** Correlation of plasma mRNA or miRNA with serum ALT, HBVDNA and HCVRNA.

Plasma biomarker	ALT	P^a^	logHBVDNA^b^	P^c^	HBsAg titre^d^	P^e^	logHCVRNA^f^	P^g^
	(correlation coefficient)		(correlation coefficient)		(correlation coefficient)		(correlation coefficient)	
Albumin mRNA	0.369	3.87E-07	0.547	1.89E-11	0.564	4.35E-12	0.426	0.003
HP mRNA	0.311	2.32E-05	0.504	1.19E-09	0.52	3.12E-10	0.408	0.005
CYP2E1 mRNA	0.31	2.51E-05	0.635	6.06E-16	0.615	1.19E-14	0.037	0.805
ApoA2 mRNA	0.372	3.18E-07	0.59	1.93E-13	0.559	6.93E-12	0.227	0.13
miRNA-122	0.204	0.006	0.436	2.37E-07	0.452	8.76E-08	-0.02	0.896

a: P value of correlation between each plasma biomarker and ALT;

b:Only subjects in HBV active and HBV carrier group were included;

c: P value of correlation between each plasma biomarker and logHBVDNA;

d: Only subjects in HBV active and HBV carrier group were included;

e: P value of correlation between each plasma biomarker and HBsAg titre;

f: Only subjects in HCV group were included;

g: P value of correlation between each plasma biomarker and logHCVRNA.

**Table 5:** Multiple linear regression analysis of plasma mRNA or miRNA with HBsAg titre, serum ALT, total bilirubin, alkaline phosphatase and γ-glutamyl transpeptidase.

Clinical Variables	Regression Coefficient	SE of Regression Coefficient	95% CI		*P* value	Multiple Regression Coefficient (R)
			Lower	Upper		
**Albumin mRNA**						**0.699**
logHBsAg	0.491	0.05	0.39	0.592	2.83E-17	
logALT	0.413	0.22	–0.021	0.847	0.062	
**miRNA-122**						**0.421**
logHBsAg	0.276	0.058	0.162	0.39	4.12E-06	

### Encapsidation of mRNA fragments and microRNA-122 in HBsAg particles

**Figure 4:**
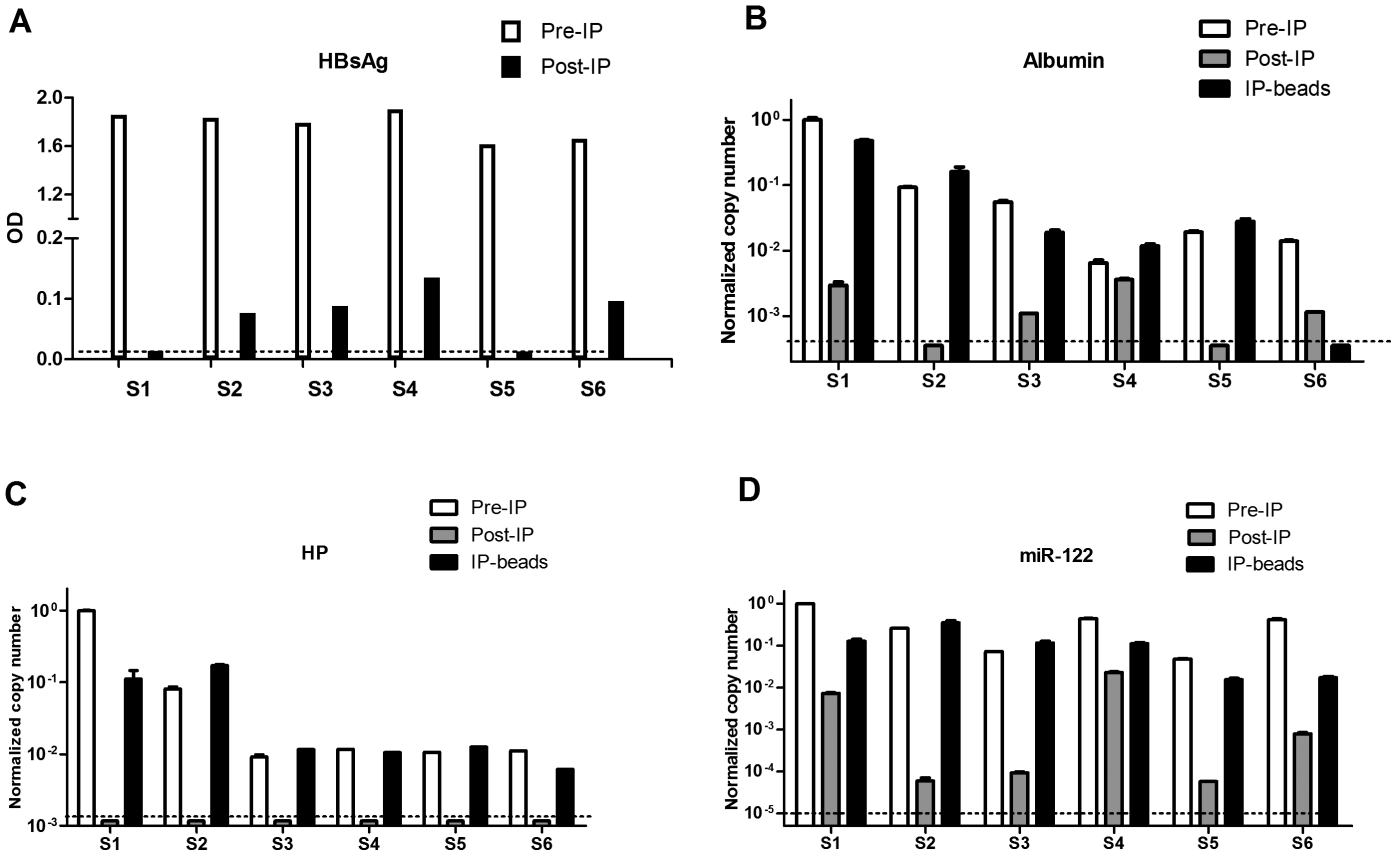
Encapsidation of mRNA fragments and microRNA-122 in HBsAg particles. (A) Relative quantification of HBsAg in Pre-IP and Post-IP supernatant. Quantification of albumin mRNA (B), HP mRNA (C) and miRNA-122 (D) in Pre-IP, Post-IP sample and precipitated beads were plotted. The dashed lines indicate limit of detection.

Since HBsAg level was found to be highly correlated with miRNA-122/mRNAs and a recent report indicated that miRNAs can associate with surface particles in patients’ plasma [11], we inferred that mRNA for albumin etc can also be encapsidated into viral or subviral particles. We performed immunoprecipitation (IP) of surface antigen from six HBsAg positive plasma samples. The relative quantification of HBsAg in Pre and Post-IP samples suggested that over 90% depletion of surface antigen after precipitation (Fig. 4A). Accordingly, a similar depletion of albumin mRNA was also observed in five of the six samples (Fig 4B). Indeed, albumin mRNA was below the limit of detection in Post-IP supernatant from sample 2 and 5. Strikingly, we were unable to detect HP mRNA in all of the six Post-IP samples whereas Pre-IP and precipitated beads contained reasonable amount of HP mRNA (Fig. 4C). A similar pattern was also found in miRNA-122 which is in accordance with published data [11].

## Discussion

Since the initial discovery of the existence of miRNAs in cell free plasma [4], [12], [13], numerous reports have identified circulating miRNA as markers for a wide range of cancer types [4], [5], [13], liver injury caused by hepatoxic agents [2], [3] or hepatitis B virus [10] etc. However, to apply circulating miRNAs into medical practice, a number of criteria should be satisfied. First, an ideal biomarker should be physiologically related to the diseased organ with a clear cause-and-effect mechanism; second, it should exhibit excellent signal-to-noise ratio compared with healthy condition and its quantification should not be easily perturbed by preanalytical and analytical variables e.g., cellular contaminants such as platelets or erythrocytes, RNA extraction method and amplification procedures [14].

For the first criterion, a recent report has raised serious concern on the possible blood cell origin of most circulating miRNAs. The authors found that, of 79 claimed miRNA markers for solid tumors, 58% are highly expressed in one or more blood cell types [15]. Furthermore, plasma level of myeloid and lymphoid miRNAs correlated closely with changes in corresponding cell counts in a patient undergoing autologous hematopoietic stem cell transplantation. This raised the possibility that many miRNAs reflected a secondary effect on blood cells rather than a tumor specific origin. For the second criterion, Macdonald et al found that the sum of intra and inter-assay variance of Cq values of selected miRNAs can reach 1.5 cycle which corresponds to over three fold difference [14]. Thus, a marginal difference between control and disease groups will be easily masked by various imprecisions introduced into the quantification protocol. Furthermore, Cheng et al found that 72% of the detectable miRNA can be substantially affected by blood processing protocol. The contamination of platelets in plasma accounts for the majority of difference [16]. Of note, miR-122 is among the few tissue specific miRNAs that are not affected by sample processing methods. Indeed, it has been repeatedly confirmed as the most reliable and sensitive circulating miRNA in drug or HBV induced liver injury. Wang et al found the miR-122 exhibited the highest fold change (>200) in plasma after acetaminophen overdose using microarray analysis [2]. Similar findings were reported by Bala et al, in which, miR-122 in plasma increased over 300 fold whereas miR-155, miR-146a or miR-125b only increased two to five fold after acetaminophen treatment [3]. In clinical HBV infection, Li et al performed comprehensive Solexa sequencing and qRT-PCR assays to search for microRNA markers of chronic hepatitis B. Again, miR-122 was reported to be the most sensitive one [10]. Thus, in this study, we chose miR-122 alone as the representative of miRNAs for comparison in HBV/HCV induced liver disease.

In recent years, evidence has also emerged that fragments of cellular messenger RNAs are also detectable in plasma/serum which can serve as indicators for various cancers [17], [18] and liver pathologies [6], [7], [8]. Nevertheless, there is a dearth of reports comparing these two categories of RNA markers in relevant clinical settings. To this end, we attempted to evaluate the abundance of circulating mRNAs (albumin, HP, CYP2E1 and APOA2) and microRNA (miRNA-122) in plasma samples of healthy volunteers and patients infected with HBV or HCV. Our results suggest that, compared with miR-122, mRNA markers enjoyed a much lower baseline in healthy condition which resulted in much higher fold change and higher statistical confidence. Furthermore, albumin mRNA can reliably discriminate HBV carriers from healthy volunteers (over 6 fold, p = 0.0006) whereas miRNA-122 showed no difference (1.1 fold, p>0.05). Intriguingly, no apparent up-regulation of miRNA-122 was observed in HCV patients even though the median sALT was three fold of the healthy control (75 v.s. 24.5 U/L). These data strongly suggest that the level of these RNA markers may not be solely determined by hepatocyte damage. Rather, the type of virus infection may strongly influence the secreted mRNA/miRNA profile. In HBV infection, multiple linear regression analysis suggests that HBsAg titre, rather than sALT accounts for the most change in plasma albumin mRNA level.

Interestingly, a recent report proposed that miRNAs can be actively secreted into HBV subviral and viral particles [11] which led us to investigate whether mRNA fragments can be encapsidated into surface particles as well. Indeed, albumin and HP mRNA was markedly depleted in Post-IP supernatant of HBsAg positive plasmas and enriched into precipitated subviral/viral particles. These data support the notion that liver specific transcripts with high abundance may take a hitchhike in the process of assembly and secretion of HBsAg particle.

Taken together, our results revealed that plasma messenger RNAs exhibited much higher sensitivity and specificity compared with miRNA-122 at least in HBV/HCV induced chronic liver diseases. The much lower baseline of these mRNA fragments in healthy condition accounts for the most of comparative advantage. In addition, a higher degree of correlation with HBsAg titre than with sALT was noted in these RNAs. Further experiments confirmed that, like microRNAs, albumin and HP mRNAs can be encapsidated by HBV viral and subviral particles. Our findings suggest that circulating, tissue specific mRNA merits more attention in the pursuit of next generation biomarkers for various maladies.

## Supporting Information

Table S1 **Correlation matrix of mRNA and microRNA marker.** The correlation coefficient (Spearman correlation) of each pair of markers were calculated and presented.
(DOCX)
