# Supplementary material for: Comparison of Circulating, Hepatocyte Specific Messenger RNA and microRNA as Biomarkers for Chronic Hepatitis B and C
Source: PLoS One. 2014 Mar 18;9(3):e92112. doi: 10.1371/journal.pone.0092112 (PMC3958472; doi:10.1371/journal.pone.0092112)
Supplement: Table S1 — Correlation matrix of mRNA and microRNA marker. The correlation coefficient (Spearman correlation) of each pair of markers were calculated and presented. (DOCX) [file pone.0092112.s001.docx]

Supporting Table 1.Correlation matrix of mRNA and microRNA markers

|  | HP | ALB | CYP2E1 | miR122 | APOA2 |
| --- | --- | --- | --- | --- | --- |
| HP | 1 | 0.858 | 0.789 | 0.736 | 0.795 |
| ALB |  | 1 | 0.81 | 0.761 | 0.746 |
| CYP2E1 |  |  | 1 | 0.674 | 0.855 |
| miR122 |  |  |  | 1 | 0.685 |
| APOA2 |  |  |  |  | 1 |
